# Multidimensional lipid and inflammatory risk control following evolocumab treatment in real-world ASCVD: a hospital-based cohort study

**DOI:** 10.3389/fphar.2026.1890158

**Published:** 2026-08-11

**Authors:** Qiang Niu, Xiaolu Liu, Hui Zhang, Qilei Wang, Bo Li

**Affiliations:** 1 Department of Cardiology, Zibo Central Hospital, Zibo, China; 2 Department of Outpatient Services, Zibo Second Cadre Sanatorium, Zibo, China

**Keywords:** ASCVD, evolocumab, LDL-C, lipid, PCSK9 inhibitor

## Abstract

**Background:**

Evolocumab lowers low-density lipoprotein cholesterol (LDL-C) and reduces cardiovascular events in patients with established atherosclerotic cardiovascular disease (ASCVD). In routine care, however, the LDL-C course is observed through irregular laboratory testing, changing background therapy, and variable follow-up. We examined lipid response, inflammatory risk control, post-index laboratory abnormalities, and exploratory risk-index outcomes after evolocumab initiation in a hospital-based ASCVD cohort.

**Methods:**

This retrospective real-world cohort used routine hospital data from ASCVD patients with recorded evolocumab exposure. Patients were classified into documented statin and no documented statin groups according to whether concomitant statin therapy was recorded in available hospital medication records during the pre-index 30-day window. Baseline lipids were defined as the patient-level median of traceable pre-index hospital laboratory measurements. The primary lipid follow-up period was 30–120 days; later periods were 121–365 days and beyond 365 days. The primary endpoint was percentage change in LDL-C at 30–120 days. LDL-C goal attainment, LDL-C reduction ≥ 50%, other lipid markers, C-reactive protein-triglyceride glucose index (CTI), inflammatory markers, longer-term LDL-C patterns, and post-index laboratory abnormalities were analyzed as secondary or exploratory outcomes. Between-group comparisons used propensity-score overlap weighting with bootstrap confidence intervals and were interpreted as adjusted observational associations.

**Results:**

Of 250 patients with recorded evolocumab exposure, 183 were included in the main analysis cohort: 137 in the documented statin group and 46 in the no documented statin group. Median LDL-C fell from 2.85 to 1.08 mmol/L, a median reduction of 63.3%. LDL-C <1.4 mmol/L was achieved in 82/117 patients (70.1%), and LDL-C reduction ≥ 50% was achieved in 81/117 patients (69.2%). In the endpoint-specific core clinical propensity model, LDL-C percentage reduction favored the documented statin group (weighted difference, −10.34 percentage points [95% CI, −20.85 to −0.045]; P = 0.048); the expanded lipid-adjusted sensitivity model remained directionally consistent but crossed the null. In the predefined exploratory CTI analysis, hsCRP-TyG Delta CTI favored the documented statin group (adjusted difference, −0.423 [95% CI, −0.749 to −0.097]; P = 0.011), whereas CRP-TyG pointed in the same direction without reaching statistical significance (−0.247 [95% CI, −0.564 to 0.071]; P = 0.128). Dual-target control was achieved in 55/131 patients (42.0%) and was more frequent in the documented statin group.

**Conclusion:**

In this hospital-based real-world ASCVD cohort, evolocumab was associated with marked LDL-C lowering in routine care. A broader pattern of lipid and exploratory residual-risk marker improvement was observed in patients with documented concomitant statin therapy. These findings are consistent with guideline-recommended lipid management, although the between-group results should be interpreted as adjusted observational associations rather than causal relationships.

## Introduction

Patients with established ASCVD remain at high risk for recurrent cardiovascular events despite contemporary lipid-lowering therapy. Current dyslipidaemia guidelines recommend intensive LDL-C reduction for very-high-risk secondary prevention patients, including both a ≥50% reduction from baseline and an LDL-C goal of <1.4 mmol/L when clinically appropriate (Mach et al., 2020). Proprotein convertase subtilisin/kexin type 9 (PCSK9) inhibition with evolocumab has shown robust LDL-C lowering and cardiovascular outcome benefit in randomized evidence (Sabatine et al., 2017). Nevertheless, clinical trial estimates do not fully describe what happens after evolocumab is introduced in routine care, where treatment persistence, background statin use, laboratory timing, follow-up frequency, and clinical decision-making may be heterogeneous.

Statin intolerance is one common reason that routine care diverges from the trial setting. Although many patients can continue some statin exposure after dose adjustment or rechallenge, a clinically meaningful subgroup cannot maintain statin therapy because of muscle symptoms, liver-enzyme concerns, perceived intolerance, or poor persistence (Cheeley et al., 2022). In such patients, PCSK9 inhibitors may be used with little or no background statin therapy. Dedicated evolocumab trials in statin-intolerant patients, including GAUSS-2 and GAUSS-3, showed substantial LDL-C lowering compared with ezetimibe, but these were short-term lipid trials rather than cardiovascular-outcome studies (Stroes et al., 2014; Nissen et al., 2016). A recent network meta-analysis of lipid-lowering regimens also suggested that PCSK9 inhibitors combined with statins provided the most favorable reduction in major cardiovascular events, whereas PCSK9 inhibitor use without background statin therapy did not show the same event signal (Niu et al., 2025). Whether patients receiving evolocumab without documented concomitant statin therapy in routine ASCVD care achieve comparable multidimensional risk-marker control remains less well described.

Real-world studies are therefore important for understanding both treatment effectiveness and treatment implementation. The European HEYMANS registry showed early and sustained LDL-C reductions over 30 months after evolocumab initiation, supporting the durability of LDL-C lowering in clinical practice (Ray et al., 2023). However, real-world lipid data still pose methodological challenges. A single post-treatment LDL-C value may reflect visit timing, short-term adherence, temporary discontinuation, intercurrent illness, or laboratory availability rather than the patient’s broader lipid trajectory.

Beyond LDL-C, residual risk assessment increasingly includes atherogenic lipid burden, Lp(a), small dense LDL-C, and inflammation. Non-HDL-C captures cholesterol in apoB-containing lipoproteins and is recommended as a secondary lipid target in guideline frameworks (Mach et al., 2020). Lp(a) is a causal ASCVD risk factor and is increasingly viewed as part of global residual risk assessment (Kronenberg et al., 2022). Small dense LDL-C is also associated with incident coronary heart disease and broader atherogenic dyslipidaemia (Liou and Kaptoge, 2020). In parallel, the residual inflammatory risk framework, commonly operationalized using hsCRP ≥2 mg/L in cardiovascular outcome trials, has been strengthened by anti-inflammatory outcome trials and related analyses (Ridker et al., 2017; Ridker et al., 2018).

We therefore conducted a hospital-based real-world study of ASCVD patients receiving evolocumab. The aims were to evaluate early and longer-term LDL-C response, compare lipid and inflammatory risk control between patients with and without documented concomitant statin therapy, examine exploratory residual risk indices, and describe post-index ALT/AST and CK laboratory abnormalities in routine care.

## Methods

### Study design and data source

This was a retrospective real-world cohort study using routine hospital clinical data collected between October 2019 and December 2024. When structured fields were incomplete, investigators reviewed available source records retained in the clinical record system and supplemented traceable information when appropriate. The study was designed and reported according to STROBE and RECORD principles for observational studies using routinely collected health data (von Elm et al., 2007; Benchimol et al., 2015). The study used routine hospital clinical data that were de-identified or anonymized before statistical analysis. The study protocol was approved by the Ethics Committee of Zibo Central Hospital; the requirement for informed consent was waived where applicable. Direct identifiers were excluded from analysis outputs.

### Study population and index date

Patients were eligible if they had ASCVD and a recorded evolocumab exposure in the hospital clinical data. ASCVD was identified from structured diagnosis and procedure records, using prespecified categories and available source-code fields for coronary artery disease or chronic ischemic heart disease, acute coronary syndrome or unstable angina, myocardial infarction, ischemic stroke or cerebral infarction, and coronary revascularization. The index date was the earliest reliable evolocumab treatment record. Patients were excluded from the main analysis cohort if pre-index LDL-C was not traceable, ASCVD-related diagnostic documentation was insufficient, or manual review identified prior PCSK9 inhibitor exposure.

### Exposure groups

Patients were assigned to the group without documented concomitant statin therapy when no concomitant statin record was documented during the 30 days before the prespecified evolocumab initiation date. Patients with a statin record during the same pre-index window were assigned to the documented concomitant statin therapy group. Accordingly, the between-group analysis was interpreted as an observational comparison of documented treatment patterns.

### Baseline and follow-up lipid definitions

Pre-index hospital laboratory results were used to define baseline LDL-C and other lipid markers. Same-day duplicate results for the same marker were summarized by their median. In the main analysis, baseline lipid values were the patient-level median of all traceable pre-index hospital measurements after this same-day deduplication step. Post-index lipids were summarized in the same way within each follow-up period, using the patient-level median when more than one value was available. This choice was made to reduce the influence of isolated laboratory values and to keep baseline and follow-up summaries aligned. To examine temporal bias and regression-to-the-mean, we also summarized the number and timing of pre-index LDL-C measurements and repeated the primary LDL-C analysis using the closest available pre-index LDL-C value within 90 days and within 30 days before index. Laboratory values were obtained from the hospital laboratory information system after unit and plausibility checks.

The primary lipid follow-up period was 30–120 days after index, approximating early post-initiation lipid reassessment. To describe later routine-care LDL-C patterns and reduce reliance on a single follow-up value, LDL-C response was also summarized in the 121–365-day and >365-day follow-up periods. All analyses reported the actual denominator available for each endpoint and follow-up period.

### Outcomes

The primary endpoint was percentage change in LDL-C during the 30–120-day follow-up period. Key secondary lipid outcomes included absolute LDL-C change, LDL-C <1.4 mmol/L, LDL-C reduction ≥ 50%, non-HDL-C, Lp(a), and small dense LDL-C. C-reactive protein-triglyceride glucose index (CTI) was treated as an exploratory inflammatory-metabolic risk index and was calculated as 0.412 x ln (CRP or hsCRP [mg/L]) + ln (triglycerides [mg/dL] x fasting glucose [mg/dL])/2 (Sun et al., 2025; Ni et al., 2026). Triglyceride and fasting glucose values recorded in mmol/L were converted to mg/dL before CTI calculation. CRP-TyG and hsCRP-TyG were analyzed separately. CTI was calculated only when triglycerides, fasting glucose, and CRP or hsCRP were available within a 72-h component window. Baseline CTI was the patient-level median of eligible pre-index CTI panels, and post-index CTI was the patient-level median of eligible panels obtained between day 30 and day 1095 after evolocumab initiation. To assess the influence of acute inflammatory states, a sensitivity analysis excluded CTI panels considered clinically suggestive of acute infection or other inflammatory conditions.

Inflammatory outcomes included dual-target control, defined as LDL-C <1.4 mmol/L plus available CRP or hsCRP <2 mg/L, and CRP or hsCRP ≥ 2 mg/L as a descriptive inflammatory-risk marker. Post-index laboratory abnormalities were defined as ALT/AST ≥ 3 × ULN and CK ≥ 3 × ULN among patients with available testing; these abnormalities were described as laboratory findings rather than adjudicated drug-related adverse reactions. Major adverse cardiovascular events were not included as a study endpoint because reliable event adjudication and death ascertainment were unavailable.

### Statistical analysis

Continuous variables were summarized as median with interquartile range and categorical variables as count and percentage. Analyses used available data for each endpoint, with denominators reported in the corresponding tables and figures. The single primary endpoint was percentage change in LDL-C from baseline to 30–120 days; other lipid, CTI, inflammatory, longer-term, and laboratory-abnormality outcomes were treated as secondary or exploratory. Between-group comparisons used propensity-score overlap weighting (Thomas et al., 2020; Li et al., 2018), with covariate balance assessed by standardized mean differences and values > 0.10 considered residual imbalance. Because treatment classification was based on available hospital medication records, these comparisons were interpreted as adjusted observational associations. Overlap-weighted differences were estimated with 2000 bootstrap 95% confidence intervals using patient-level resampling and re-estimation of propensity scores and weights. For binary outcomes, risk differences were expressed in percentage points. Assay-specific Delta CTI was defined as post-index median CTI minus baseline median CTI and evaluated using ANCOVA adjusted for baseline CTI, age, sex, diabetes, and baseline LDL-C, with an additional timing sensitivity model adjusting for median post-index CTI measurement day. P values were two-sided; the primary endpoint was prespecified, whereas secondary and exploratory P values were nominal and hypothesis-generating. To assess the influence of baseline timing, baseline LDL-C availability was summarized and the primary LDL-C analysis was repeated using the closest available pre-index LDL-C value within 90 and 30 days before index.

Follow-up laboratory testing reflected routine care rather than a fixed protocol. Analyses were therefore endpoint-specific complete-case analyses, with denominators reported for each endpoint. To assess whether laboratory availability was informative, patients with and without an available 30–120-day LDL-C measurement were compared, and an inverse-probability-of-observation weighted sensitivity analysis was performed for the primary LDL-C percentage-change endpoint. Longer-term LDL-C patterns were additionally examined using a repeated-measures Gaussian generalized estimating equation model with an exchangeable working correlation across the 30–120-day, 121–365-day, and >365-day windows, including treatment group, follow-up window, their interaction, baseline LDL-C, age, and sex.

## Results

### Cohort and baseline characteristics

**FIGURE 1:**
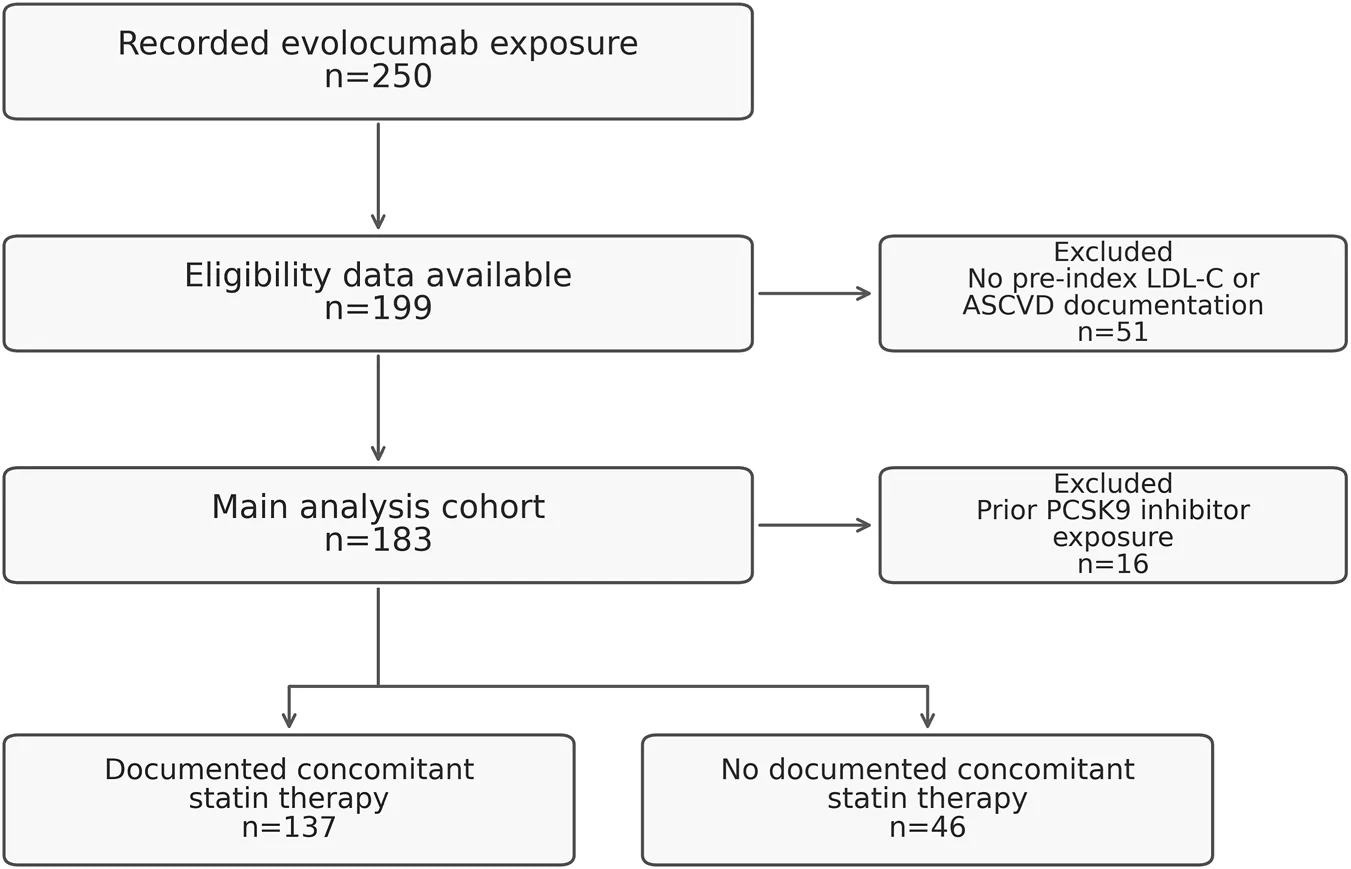
Cohort construction and exclusion reasons.

Of 250 patients with recorded evolocumab exposure in the hospital clinical data, 51 were excluded because pre-index LDL-C was not traceable and/or ASCVD-related diagnostic documentation was insufficient. Among the remaining 199 patients, 16 were excluded after manual review because prior PCSK9 inhibitor exposure was identified. The final cohort included 183 patients: 137 with documented concomitant statin therapy and 46 without documented concomitant statin therapy. Median age was 60 years, and 115 patients (62.8%) were male. Clear coronary artery disease diagnosis records were present in 163 patients (89.1%). Median baseline LDL-C was 2.85 (2.34–3.54), and median baseline Lp(a) was 120 (64.3–438). In the expanded propensity model, the maximum absolute standardized mean difference in the full cohort decreased from 0.442 before weighting to 0.016 after overlap weighting. Endpoint-specific balance diagnostics also showed maximum weighted standardized mean differences of 0.032–0.033 for the overlap-weighted lipid and inflammatory analyses. The cohort flow and baseline characteristics are shown in Figure 1 and Table 1. Baseline characteristics of included and excluded patients are summarized in Supplementary Material.

**TABLE 1:** Baseline characteristics and covariate balance before and after overlap weighting.

Characteristic	Overall (N = 183)	Documented statin (n = 137)	No documented statin (n = 46)	Unweighted SMD	Overlap-weighted SMD
Age, years	60 (60–70)	60 (60–70)	60 (50–70)	0.312	0.013
Male sex	115 (62.8%)	89 (65.0%)	26 (56.5%)	0.174	0.012
Coronary artery disease-related evidence	163 (89.1%)	127 (92.7%)	36 (78.3%)	0.419	0.009
ACS/unstable angina	87 (47.5%)	64 (46.7%)	23 (50.0%)	0.066	0.015
Myocardial infarction history	48 (26.2%)	36 (26.3%)	12 (26.1%)	0.004	0.011
Stroke history	44 (24.0%)	30 (21.9%)	14 (30.4%)	0.195	0.005
Hypertension	94 (51.4%)	68 (49.6%)	26 (56.5%)	0.138	0.007
Diabetes	58 (31.7%)	45 (32.8%)	13 (28.3%)	0.100	0.003
Chronic kidney disease	12 (6.6%)	9 (6.6%)	3 (6.5%)	0.002	0.000
Heart failure	40 (21.9%)	32 (23.4%)	8 (17.4%)	0.149	0.006
Prior coronary angiography/PCI/CABG	125 (68.3%)	99 (72.3%)	26 (56.5%)	0.333	0.007
Ezetimibe record near index	38 (20.8%)	34 (24.8%)	4 (8.7%)	0.442	0.016
LDL-C, mmol/L	2.85 (2.34–3.54)	2.73 (2.28–3.5)	2.99 (2.46–3.75)	0.162	0.000
Non-HDL-C, mmol/L	3.31 (2.81–4.31)	3.27 (2.75–4.11)	3.78 (3.02–4.43)	0.275	0.016
Lp(a), mg/L	120 (64–438)	117 (67–458)	165 (52–418)	0.134	0.004
sdLDL-C, mmol/L	0.82 (0.58–1.05)	0.82 (0.57–1.05)	0.87 (0.62–1.07)	0.058	0.013

Values are median (IQR) or n (%). Documented statin indicates documented concomitant statin therapy during the pre-index window. SMD, standardized mean difference.

The median number of pre-index LDL-C measurements per patient was 2 (IQR, 1–5), and 121/183 patients (66.1%) had at least two pre-index LDL-C measurements. The closest pre-index LDL-C measurement was obtained a median of 5 days (IQR, 2–29) before the index date. A pre-index LDL-C value was available within 30 days in 137/183 patients (74.9%), within 90 days in 159/183 (86.9%), and within 365 days in 172/183 (94.0%).

### Primary and longer-term LDL-C outcomes

In the primary 30–120-day follow-up period, 117 patients had paired LDL-C data. LDL-C decreased from 2.85 (2.36–3.75) mmol/L at baseline to 1.08 (0.7–1.57) mmol/L after evolocumab initiation, with a median percentage reduction of 63.3% (IQR, 42.4%–78.4%). LDL-C <1.4 mmol/L was achieved in 82/117 patients (70.1%), and LDL-C reduction ≥50% was observed in 81/117 patients (69.2%). Primary LDL-C estimates are summarized in Table 2. The primary LDL-C finding was consistent in baseline-timing sensitivity analyses using the closest available pre-index LDL-C value within 90 days and within 30 days, with median percentage reductions of 63.6% (IQR, 38.1%–75.6%; n = 107) and 63.9% (IQR, 39.0%–75.4%; n = 97), respectively.

**TABLE 2:** Main lipid and exploratory CTI outcomes after evolocumab initiation.

Endpoint	Window	N	Baseline	Follow-up	Absolute change	Percent change
LDL-C, mmol/L	30–120 days	117	2.85 (2.36–3.75)	1.08 (0.7–1.57)	−1.63 (−2.48 to −1.09)	−63.3% (−78.4% to −42.4%)
Non-HDL-C, mmol/L	30–120 days	117	3.46 (2.81–4.34)	1.33 (0.9–1.98)	−1.83 (−2.88 to −1.3)	−63.1% (−74.8% to −42.9%)
Lp(a), mg/L	30–120 days	117	121 (65–418)	67.5 (35.5–341)	−29 (−87 to −0.3)	−29.3% (−57.9% to −0.8%)
sdLDL-C, mmol/L	30–120 days	116	0.82 (0.6–1.1)	0.33 (0.21–0.51)	−0.4 (−0.66 to −0.24)	−56.4% (−73.7% to −38.8%)
hsCRP-TyG index	30–1095 days; 72 h window	51	4.995 (4.645–5.462)	4.664 (4.262–5.163)	−0.123 (−0.638 to 0.161)	Not reported
CRP-TyG index	30–1095 days; 72 h window	81	4.796 (4.469–5.514)	4.737 (4.279–5.153)	−0.226 (−0.565 to 0.244)	Not reported

Values are median (IQR) among patients with paired data. CTI, C-reactive protein-triglyceride glucose index; components were measured within 72 h.

In the 30–120-day endpoint-specific overlap-weighted analysis, patients with documented concomitant statin therapy had a greater LDL-C percentage reduction than those without documented statin therapy (adjusted difference, −10.34 percentage points [95% CI, −20.85 to −0.045]; P = 0.048). They were also more likely to achieve LDL-C <1.4 mmol/L (adjusted risk difference, 24.7 percentage points [95% CI, 3.63 to 46.6]; P = 0.021) and LDL-C reduction ≥50% (adjusted risk difference, 24.2 percentage points [95% CI, 3.37 to 44.5]; P = 0.024). Additional balance, sensitivity, and missingness analyses are reported in the Supplementary Material and were consistent with the primary LDL-C comparison.

### Longer-term follow-up analysis of LDL-C response

**FIGURE 2:**
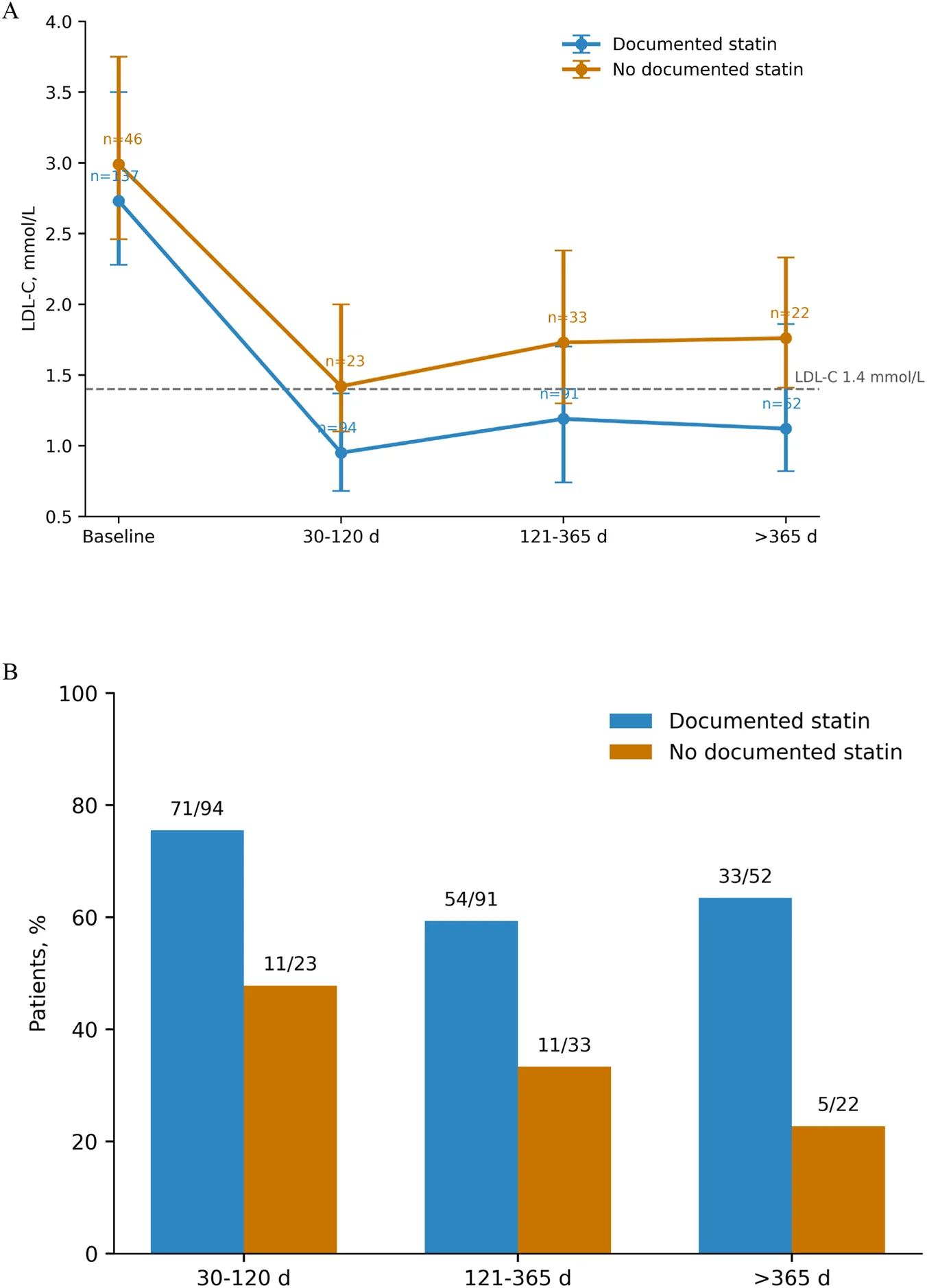
**(A)** LDL-C from baseline through follow-up periods. **(B)** LDL-C goal attainment across follow-up periods.

Because follow-up lipid testing was not protocolized in routine care, later LDL-C windows are presented as available-case observed responses. Among patients with paired measurements, median LDL-C decreased from 2.79 to 1.33 mmol/L at 121–365 days (n = 124; median percentage reduction, 53.3% [IQR, 31.0%–70.1%]) and from 2.84 to 1.37 mmol/L beyond 365 days (n = 74; 51.8% [IQR, 36.5%–64.0%]). LDL-C goal attainment remained more frequent in the documented statin group in these later windows: 54/91 (59.3%) versus 11/33 (33.3%) at 121–365 days and 33/52 (63.5%) versus 5/22 (22.7%) beyond 365 days. These longer-term LDL-C summaries are shown in Figures 2A,B, and Table 3.

**TABLE 3:** LDL-C response and goal attainment across clinically defined follow-up periods.

Window	Paired N	Follow-up LDL-C	LDL-C percent change	LDL-C <1.4 mmol/L	Follow-up day
30–120 days	117	1.08 (0.7–1.57)	Overall: 63.3% (−78.4% to −42.4%) Documented statin: 67% (−79.7% to −47.2%) No documented statin: 44.8% (−62.9% to −33.7%)	Documented statin: 71/94 (75.5%) No documented statin: 11/23 (47.8%)	66.9 (47.5–84.4)
121–365 days	124	1.33 (0.81–1.9)	Overall: 53.3% (−70.1% to −31%) Documented statin: 58.5% (−74.2% to −40.5%) No documented statin: 41.9% (−56.6% to −23.2%)	Documented statin: 54/91 (59.3%) No documented statin: 11/33 (33.3%)	231 (193–273)
>365 days	74	1.37 (0.91–2)	Overall: 51.8% (−64% to −36.5%) Documented statin: 56.8% (−69.3% to −39.9%) No documented statin: 43.9% (−54% to −33.2%)	Documented statin: 33/52 (63.5%) No documented statin: 5/22 (22.7%)	505 (429–585)

Values are median (IQR) or n/N (%). Later follow-up periods are available-case summaries. LDL-C, low-density lipoprotein cholesterol.

In a repeated-measures GEE sensitivity analysis using all available LDL-C follow-up windows, LDL-C percentage reduction remained greater in the documented statin group at the 30–120-day reference window (adjusted difference, −14.27 percentage points; 95% CI, −24.09 to −4.45), with no clear evidence that the between-group pattern differed across later follow-up periods. Among patients with LDL-C data available in all three follow-up windows, LDL-C remained below baseline throughout follow-up. Details are provided in the Supplementary Material.

**FIGURE 3:**
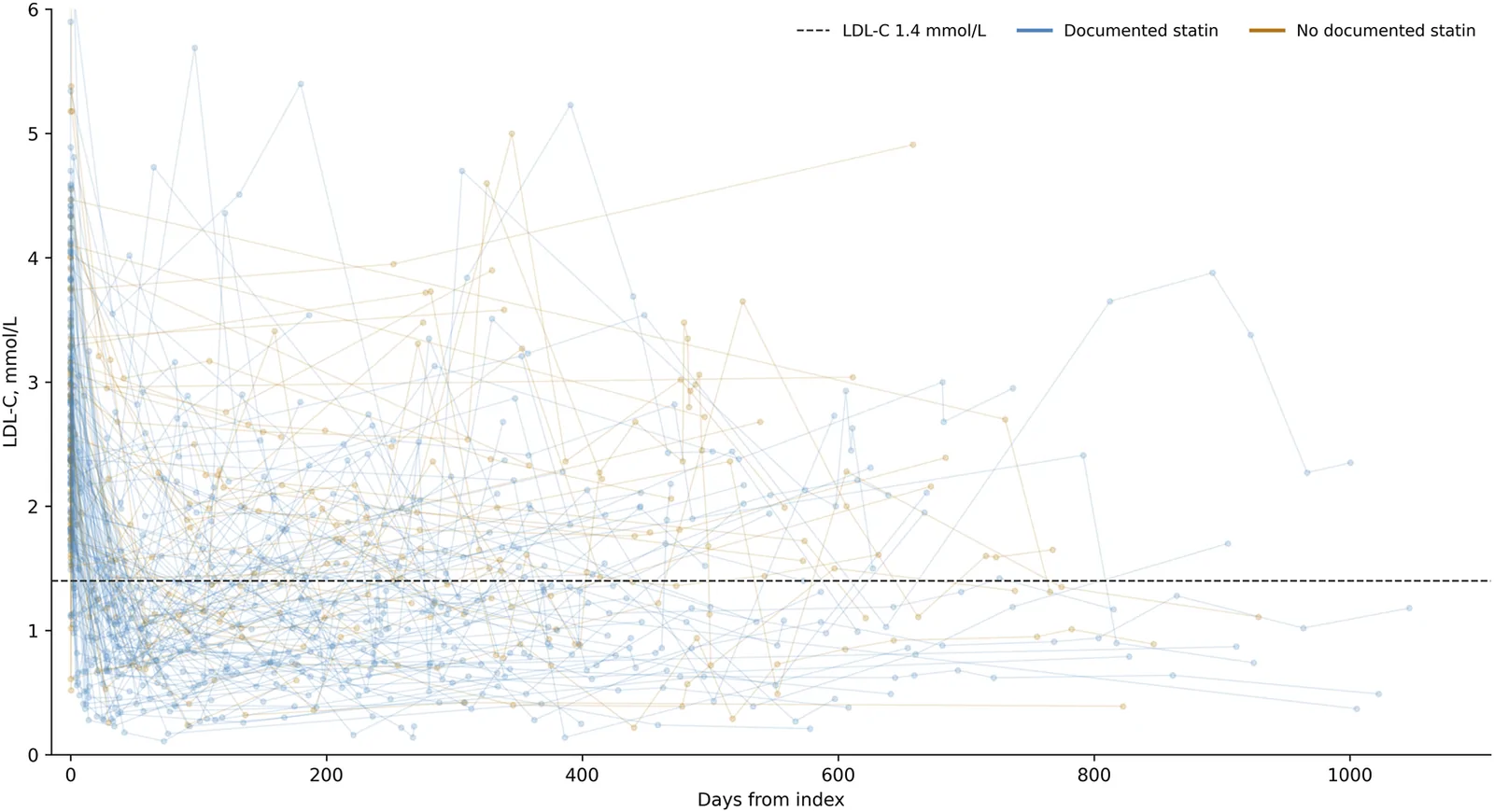
Individual LDL-C trajectories during routine follow-up.

To make the longitudinal pattern more transparent, we also plotted individual LDL-C trajectories during follow-up (Figure 3).

### Other lipid and exploratory CTI outcomes

**FIGURE 4:**
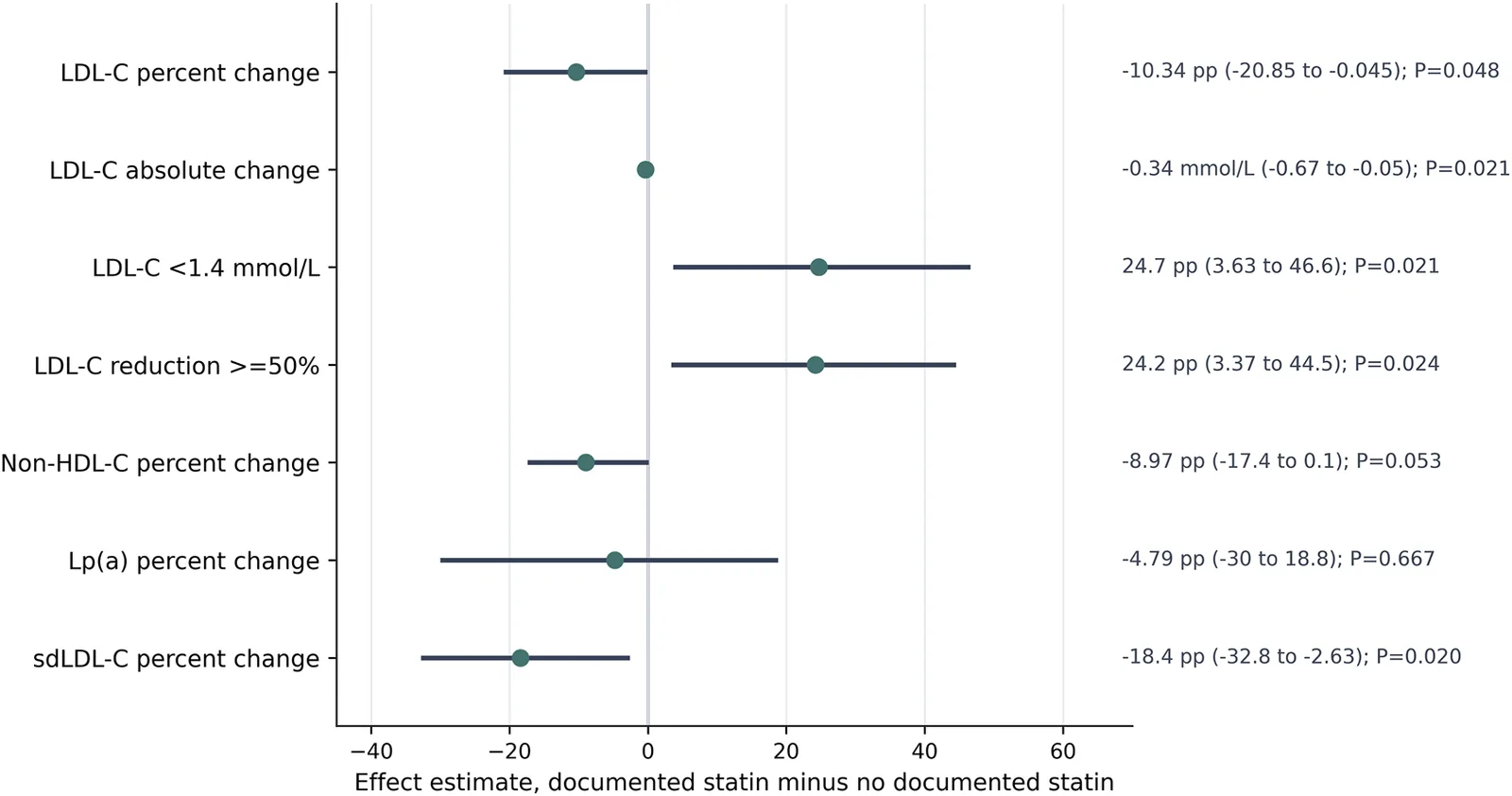
Adjusted forest plot of LDL-C and other lipid outcomes.

Secondary and exploratory outcomes were interpreted using nominal P values together with effect sizes, confidence intervals, and clinical coherence. During the same 30–120-day period, non-HDL-C, Lp(a), and sdLDL-C also decreased after evolocumab initiation, with median percentage changes of −63.1% (IQR, −74.8% to −42.9%), −29.3% (IQR, −57.9% to −0.8%), and −56.4% (IQR, −73.7% to −38.8%), respectively. In adjusted between-group comparisons, sdLDL-C percentage reduction was greater in the documented statin group (adjusted difference, −18.4 percentage points; 95% CI, −32.8 to −2.63; P = 0.020). Non-HDL-C showed a similar direction, although the confidence interval narrowly crossed the null (adjusted difference, −8.97 percentage points; 95% CI, −17.4 to 0.1; P = 0.053), whereas Lp(a) showed no clear between-group difference (adjusted difference, −4.79 percentage points; 95% CI, −30.0 to 18.8; P = 0.667). These lipid comparisons are summarized in Table 4 and Figure 4.

**TABLE 4:** Adjusted between-group comparisons for lipid and exploratory CTI outcomes.

Outcome	Window	N, documented/no documented	Documented statin	No documented statin	Adjusted difference (95% CI); P value
LDL-C percent change	30–120 days	94/23	−59.6%	−49.3%	−10.34 pp (−20.85 to −0.045); P = 0.048
LDL-C absolute change	30–120 days	94/23	−1.93 mmol/L	−1.59 mmol/L	−0.34 mmol/L (−0.67 to −0.05); P = 0.021
LDL-C <1.4 mmol/L	30–120 days	94/23	74.4%	49.7%	24.7 pp (3.63 to 46.6); P = 0.021
LDL-C reduction ≥ 50%	30–120 days	94/23	75.5%	51.3%	24.2 pp (3.37 to 44.5); P = 0.024
Non-HDL-C percent change	30–120 days	94/23	−58.0%	−49.0%	−8.97 pp (−17.4 to 0.1); P = 0.053
Lp(a) percent change	30–120 days	94/23	−17.1%	−12.3%	−4.79 pp (−30 to 18.8); P = 0.667
sdLDL-C percent change	30–120 days	94/22	−53.8%	−35.5%	−18.4 pp (−32.8 to −2.63); P = 0.020
hsCRP-TyG delta CTI (ANCOVA)	30–1095 days; 72 h window	35/16	−0.393 (−0.856 to 0.099)	0.127 (−0.156 to 0.225)	−0.423 (−0.749 to −0.097); P = 0.011
hsCRP-TyG delta CTI; acute inflammation-excluded sensitivity	30–1095 days; 72 h window	35/15	−0.393 (−0.856 to 0.099)	0.121 (−0.195 to 0.240)	−0.339 (−0.640 to −0.039); P = 0.027
CRP-TyG delta CTI (ANCOVA)	30–1095 days; 72 h window	61/20	−0.298 (−0.656 to 0.081)	0.040 (−0.422 to 0.347)	−0.247 (−0.564 to 0.071); P = 0.128
CRP-TyG delta CTI; acute inflammation-excluded sensitivity	30–1095 days; 72 h window	60/20	−0.311 (−0.659 to 0.050)	0.040 (−0.372 to 0.347)	−0.307 (−0.600 to −0.015); P = 0.039

Lipid outcomes used overlap weighting; CTI, outcomes used ANCOVA., Adjusted differences are documented statin minus no documented statin. Binary outcomes are risk differences. CTI, C-reactive protein-triglyceride glucose index; CI, confidence interval; pp, percentage points.

**FIGURE 5:**
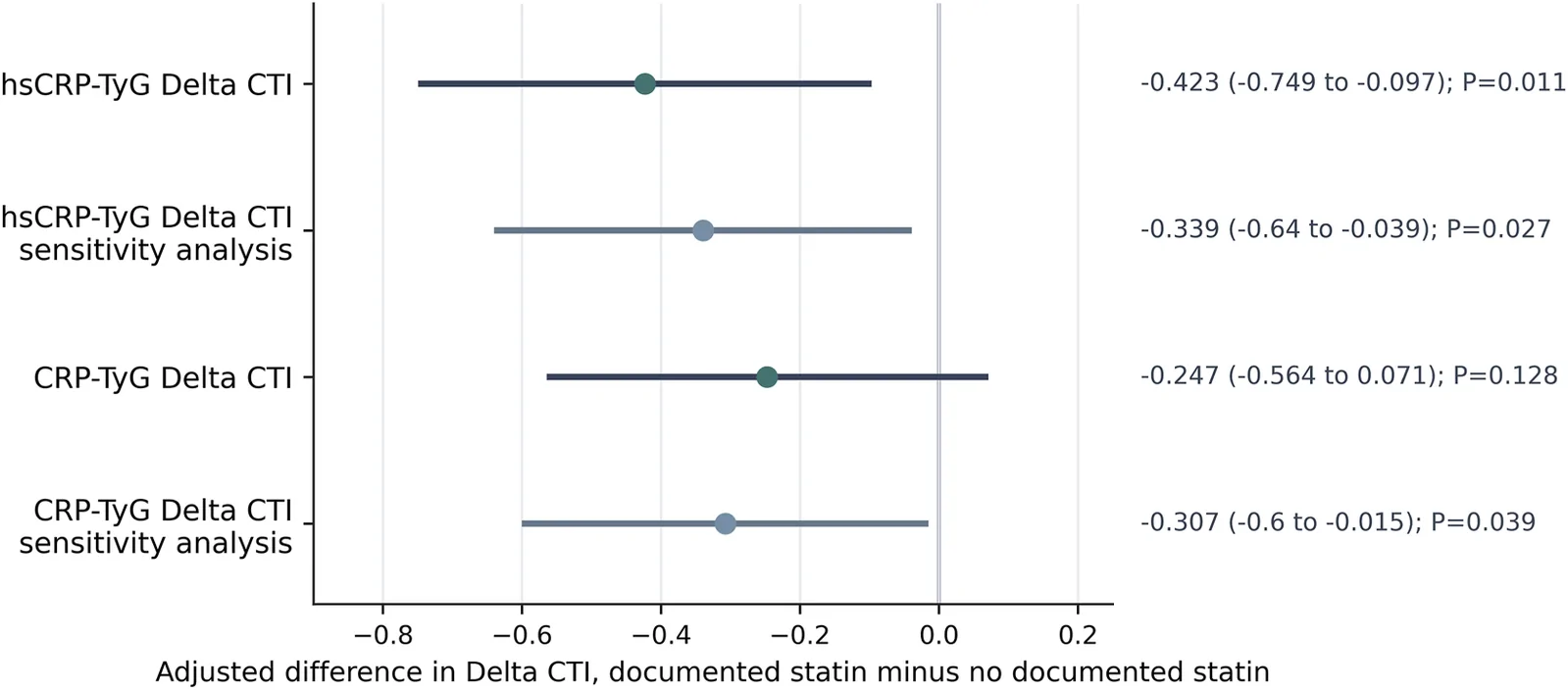
Exploratory forest plot of CTI change.

Exploratory CTI analyses were performed separately for hsCRP-TyG and CRP-TyG using a 72-h component window, with post-index panels restricted to 30–1095 days after evolocumab initiation. Among patients with eligible paired hsCRP-TyG panels, CTI decreased in the documented statin group (−0.393; IQR, −0.856 to 0.099), whereas no comparable reduction was observed in the no documented statin group (0.127; IQR, −0.156–0.225). In an ANCOVA model adjusted for baseline CTI, age, sex, diabetes, and baseline LDL-C, Delta CTI favored the documented statin group (adjusted difference, −0.423; 95% CI, −0.749 to −0.097; P = 0.011). CRP-TyG showed a similar direction with less precision; the adjusted between-group difference in Delta CTI was −0.247 (95% CI, −0.564 to 0.071; P = 0.128). In sensitivity analyses excluding measurements considered likely to reflect acute infection or other inflammatory conditions, the adjusted differences remained favorable for the documented statin group for both hsCRP-TyG (−0.339; 95% CI, −0.640 to −0.039; P = 0.027) and CRP-TyG (−0.307; 95% CI, −0.600 to −0.015; P = 0.039). Additional timing-adjusted models are provided in the Supplementary Material. Given the assay-specific subsets and the extended routine-care follow-up window, these CTI findings were interpreted as exploratory. CTI results are shown in Table 4 and Figure 5.

### Residual inflammatory risk and dual-target control

**FIGURE 6:**
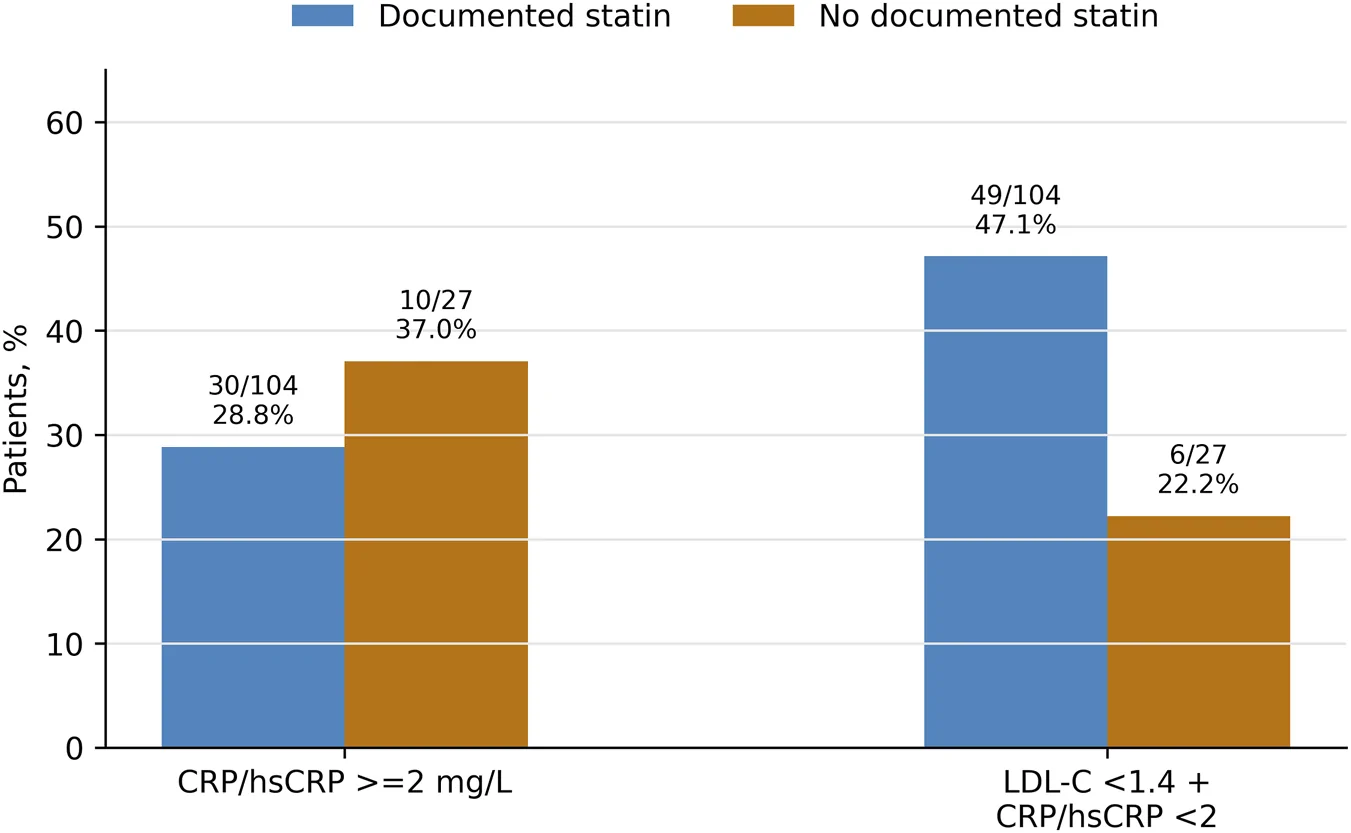
Residual inflammatory risk and dual lipid-inflammatory target control.

Exploratory dual-target control, defined as LDL-C <1.4 mmol/L together with CRP/hsCRP <2 mg/L, was observed in 55 of 131 patients (42.0%) during all post-index follow-up. The proportion was higher in the documented statin group than in the no documented statin group (49/104 [47.1%] vs. 6/27 [22.2%]; P = 0.029). After overlap weighting, the documented statin group had an 18.4-percentage-point higher absolute probability of dual-target control than the no documented statin group (95% CI, 2.21–35.0). During all post-index follow-up, CRP/hsCRP ≥ 2 mg/L was observed in 40 of 131 patients (30.5%). These results are shown in Figure 6.

### Post-index laboratory abnormalities

Post-index ALT/AST ≥ 3 x ULN occurred in 13/165 tested patients (7.9%), including 10/124 (8.1%) in the documented statin group and 3/41 (7.3%) in the no documented statin group. CK ≥ 3 x ULN occurred in 4/79 tested patients (5.1%), including 4/62 (6.5%) and 0/17 (0.0%) tested patients in the two groups, respectively. These findings should be interpreted as descriptive post-index laboratory abnormalities among tested patients, rather than adjudicated evolocumab-related adverse reactions or evidence of comparable safety. Detailed laboratory-abnormality summaries are provided in Supplementary Material.

## Discussion

### Principal findings

In this hospital-based real-world cohort of ASCVD patients receiving evolocumab, LDL-C lowering was substantial in routine care and remained evident among patients with later follow-up measurements. Non-HDL-C, Lp(a), and small dense LDL-C also declined, suggesting broader improvement in lipid-related risk markers in clinical practice. Patients with documented concomitant statin therapy showed a more favorable observed response pattern, including deeper LDL-C reduction, higher LDL-C goal attainment, more frequent lipid-inflammatory dual-target control, and a favorable exploratory hsCRP-TyG CTI signal with directionally consistent CRP-TyG findings. Post-index ALT/AST and CK abnormalities were uncommon among tested patients, but these laboratory findings should not be interpreted as adjudicated adverse reactions or as evidence of comparable safety.

The clinical message is practical rather than causal. Evolocumab remains an effective intensive lipid-lowering therapy, including in patients without documented concomitant statin use. However, the broader residual-risk marker profile appeared less complete in that group, particularly for inflammatory-metabolic markers. These findings are consistent with guideline-recommended lipid management, in which statins remain an important background therapy when tolerated and PCSK9 inhibition is used to achieve more intensive LDL-C lowering in selected high-risk patients.

### Comparison with trials and real-world evidence

The magnitude of LDL-C reduction in this cohort is consistent with the established efficacy of evolocumab in randomized and observational evidence. FOURIER demonstrated that evolocumab added to statin therapy reduced LDL-C and cardiovascular events in patients with established ASCVD (Sabatine et al., 2017). The European HEYMANS registry similarly reported early LDL-C lowering, with a median 58% reduction within 3 months and sustained reductions over 30 months in routine clinical practice (Ray et al., 2023). Our 30–120-day follow-up period reduction of 63.3% is directionally consistent with these data, although direct comparison requires caution because our study used routine hospital laboratory testing, clinical-record-based index reconstruction, clinician-reviewed data consistency checks, and a nonrandomized treatment-pattern comparison.

The higher LDL-C goal attainment observed among patients with documented concomitant statin therapy also fits the clinical logic of guideline-directed lipid management. Current prevention frameworks emphasize intensive LDL-C reduction in very-high-risk ASCVD patients, often requiring combination lipid-lowering therapy (Mach et al., 2020). This interpretation is also consistent with recent comparative evidence suggesting that PCSK9 inhibitor plus statin regimens rank favorably for major cardiovascular event reduction (Niu et al., 2025). In this study, however, the group comparison should be interpreted as an adjusted observational association rather than a causal estimate of statin add-on efficacy. Treatment pattern, clinical indication, adherence, physician preference, and medication capture may all influence both the exposure group and the observed lipid response.

### Longer-term LDL-C patterns and variability

The follow-up period analysis addresses a central problem in real-world lipid studies: follow-up lipid values are not obtained according to a fixed protocol. A single follow-up LDL-C value can overrepresent one clinical moment and underrepresent the broader treatment trajectory. By summarizing patient-level median LDL-C within 30–120 days, 121–365 days, and beyond 365 days, this analysis separates early treatment response from longer-term lipid control under routine-care conditions.

The time-window pattern was clinically plausible. LDL-C fell most sharply during the early follow-up period and remained substantially lower during later follow-up, but the magnitude of reduction was modestly attenuated beyond 120 days. This should not be interpreted as loss of pharmacologic efficacy. In routine practice, longer-term LDL-C values are shaped by medication persistence, injection timing, prescription renewal, affordability, changes in background lipid-lowering therapy, intercurrent illness, and selective laboratory retesting. This supports using 30–120 days as the primary efficacy follow-up period, while presenting longer-term periods as observed durability patterns rather than protocolized durability analyses.

The individual LDL-C trajectories provide a concrete view of the same issue: most patients moved downward after treatment initiation, but the subsequent course was uneven. This is typical of routine lipid management and supports continued follow-up, attention to treatment continuity, and caution when interpreting an isolated LDL-C result.

### Residual lipid risk beyond LDL-C

A strength of this analysis is that it does not stop at LDL-C. Non-HDL-C captures cholesterol carried by apoB-containing particles and is recommended as a secondary target in guideline frameworks (Mach et al., 2020). Lp(a) is a causal ASCVD risk factor, and the 2022 EAS consensus statement recommends at least once-in-adulthood measurement and intensified management of global risk in patients with elevated Lp(a) (Kronenberg et al., 2022). Small dense LDL-C reflects an atherogenic lipoprotein phenotype and has been associated with incident coronary heart disease and ASCVD events (Liou and Kaptoge, 2020).

### Residual inflammation and dual-target control

Residual inflammatory risk is now part of secondary-prevention thinking. CANTOS enrolled patients with prior myocardial infarction and hsCRP ≥ 2 mg/L and showed that targeting inflammation could reduce recurrent cardiovascular events independent of lipid lowering (Ridker et al., 2017). A secondary CANTOS analysis linked achieved hsCRP reduction to event reduction, supporting the clinical relevance of the 2 mg/L threshold (Ridker et al., 2018). In our cohort, one clinically interpretable exploratory inflammation-related finding was dual-target control, defined as LDL-C <1.4 mmol/L plus CRP/hsCRP <2 mg/L. This endpoint keeps the focus on intensive cholesterol lowering while acknowledging that inflammatory status helps describe residual risk in high-risk ASCVD. Dual-target control was more frequently observed among patients with documented concomitant statin therapy, suggesting that documented concomitant statin therapy was associated with a more favorable exploratory laboratory-marker pattern across both lipid and inflammatory domains.

The CTI findings extend this observation from a threshold-based endpoint to an exploratory inflammatory-metabolic index. CTI was added not as a substitute for clinical events, but to ask whether an inflammatory-metabolic signal changed alongside lipid lowering. The index combines CRP or hsCRP with the triglyceride-glucose construct, so it reflects residual inflammation, insulin resistance, and metabolic stress more directly than a purely LDL-C-based marker. In the predefined 30–1095-day analysis using a 72-h component window, hsCRP-TyG decreased in the documented statin group, whereas no comparable reduction was seen in the no documented statin group; CRP-TyG pointed in the same direction with weaker precision. Results were similar in the sensitivity analysis excluding measurements considered likely to reflect acute infection or other inflammatory conditions. These findings remain exploratory because CTI is assay-specific, fluctuates with inflammatory and metabolic state, and was available only in selected patients with the required laboratory data. Still, the observed direction is biologically plausible. Evolocumab can lower LDL-C substantially without documented statin therapy, but that does not mean it reproduces the broader vascular and inflammatory effects associated with statins. FOURIER established evolocumab outcome benefit largely on background statin therapy (Sabatine et al., 2017). CANTOS showed inflammation as a separate therapeutic axis in secondary prevention (Ridker et al., 2017; Ridker et al., 2018), JUPITER supported the relevance of statin therapy in patients selected by inflammatory risk (Ridker et al., 2008), and mechanistic work has described statin-related effects on inflammatory activity, endothelial function, and plaque biology beyond LDL-C lowering (Davignon, 2004). Read this way, the CTI signal is hypothesis-generating and clinically plausible: PCSK9 inhibition may produce substantial LDL-C lowering, while documented background statin therapy was associated with a more favorable residual-risk marker pattern in this cohort.

### Post-index laboratory abnormalities

Post-index ALT/AST and CK abnormalities were uncommon among tested patients. Because testing was clinically driven and symptoms, repeat confirmation, treatment interruption, and causality adjudication were not consistently available, these findings should be interpreted as descriptive laboratory abnormalities rather than adjudicated drug-related adverse events.

### Methodological implications

Several methodological choices were important for interpreting these data. First, defining time zero from the earliest reliable evolocumab treatment record helped align lipid measurements with treatment initiation. Second, using each patient’s median pre-index lipid value reduced the influence of isolated laboratory results and made the baseline definition consistent with the post-index window summaries. Third, overlap weighting improved measured covariate balance and allowed group comparisons to be presented more transparently. These choices cannot remove all bias, but they make the timing of treatment and lipid response easier to interpret in a clinically messy dataset.

### Limitations

This study has limitations. It was retrospective and used routine hospital clinical data from a limited real-world cohort. Medication exposure capture may be incomplete, and residual confounding remains possible despite overlap weighting and ANCOVA adjustment. Lipid measurements were obtained from routine hospital laboratory records; the timing of testing reflected clinical care rather than a study-mandated visit schedule. CTI analyses were exploratory and limited by smaller assay-specific subsets, the extended 30–1095-day routine-care follow-up window, and sensitivity to acute inflammatory-metabolic measurements. Dual-target analyses were also exploratory and depended on available clinically driven LDL-C and CRP/hsCRP testing. Because inclusion required traceable pre-index LDL-C and ASCVD documentation, selection related to hospital data completeness cannot be excluded. Only one primary endpoint was specified; secondary and exploratory comparisons were not multiplicity-adjusted and were treated as supportive rather than confirmatory. Major adverse cardiovascular events were not included as a primary outcome because reliable event adjudication and death ascertainment were unavailable. Stable outpatient versus acute inpatient laboratory context could not be uniformly adjudicated for every historical measurement, and acute inflammatory conditions may still have influenced some CRP/hsCRP-based analyses despite sensitivity review.

Follow-up testing was clinically driven, so later-window lipid results and laboratory endpoints should be interpreted as available-case routine-care findings rather than protocolized durability assessments or laboratory-abnormality rates.

## Conclusion

In this real-world ASCVD cohort, evolocumab was associated with marked early LDL-C lowering, with lower LDL-C levels also observed during later available follow-up. Patients with documented concomitant statin therapy showed a broader pattern of lipid and exploratory residual-risk marker improvement, including greater LDL-C reduction, better goal attainment, greater CTI reduction in the analyzable inflammatory-metabolic subset, and higher dual lipid-inflammatory target control. These findings are consistent with guideline-recommended lipid management. Because this was an observational analysis based on available hospital medication records, the between-group results should be interpreted as adjusted observational associations rather than causal relationships. The visible LDL-C variability also highlights the importance of treatment continuity and follow-up in routine PCSK9-based management.

## Data Availability

The datasets presented in this article are not readily available because the raw hospital clinical data cannot be made publicly available because they originate from routine electronic medical records and may contain potentially identifiable patient information. Access to de-identified analysis tables and statistical code may be considered upon reasonable request to the corresponding authors, subject to institutional policies and ethics approval. Requests to access the datasets should be directed to Bo Li, libosubmit@163.com.
