# Supplementary material for: Multidimensional lipid and inflammatory risk control following evolocumab treatment in real-world ASCVD: a hospital-based cohort study
Source: Front Pharmacol. 2026 Aug 11;17:1890158. doi: 10.3389/fphar.2026.1890158 (PMC13503166; doi:10.3389/fphar.2026.1890158)
Supplement: Supplementary file 1 [file Supplementaryfile1.docx]

**Supplementary Material**

Hospital-based real-world analysis of evolocumab in patients with ASCVD

# Supplementary Methods

## Operational Eligibility and ASCVD Definition

ASCVD-related evidence was defined by any of the following before or at the index date:

• Coronary disease terms, including coronary heart disease and chronic ischemic heart disease.

• Acute coronary syndrome or unstable angina terms.

• Myocardial infarction terms.

• Ischemic cerebrovascular disease terms, including ischemic stroke, cerebral infarction, cerebral embolism/infarction, and stroke-related diagnosis text.

• Coronary procedure evidence: PCI/stent-related procedures, balloon angioplasty, thrombus aspiration, rotational atherectomy, CABG, or coronary bypass-related terms.

Where ICD-10-style source codes were available, the prespecified mapping corresponded mainly to the following ASCVD domains:

Coronary artery disease / chronic ischemic heart disease: I20, I25 and related source-code variants.

Acute myocardial infarction or recurrent myocardial infarction: I21-I22 and related source-code variants.

Ischemic stroke / cerebral infarction and occlusive cerebrovascular disease: I63, I65-I66 and related source-code variants.

History or status after coronary revascularization, when captured as diagnosis/status codes: Z95.1, Z95.5 and related source-code variants.

Procedure-based ASCVD evidence was identified from available structured procedure source-code fields and procedure names, including coronary angiography, PCI, coronary stenting, balloon angioplasty, thrombus aspiration, rotational atherectomy, CABG, and coronary bypass-related procedures.

# Supplementary Tables

**Supplementary Table S1. Baseline characteristics of included and excluded patients**

| Characteristic | Included (n=183) | Excluded (n=67) | SMD |
| --- | --- | --- | --- |
| Age, years | 60 (60 to 70) | 60 (50 to 60) | 0.476 |
| Eligibility-stage traceable LDL-C among patients with available values, mmol/L | 3.21 (2.54 to 4.03) | 1.84 (1.48 to 2.23) | 1.8 |
| Male sex | 115/183 (62.8%) | 42/67 (62.7%) | 0.003 |
| Coronary artery disease-related evidence | 163/183 (89.1%) | 32/67 (47.8%) | 0.992 |
| Myocardial infarction history | 48/183 (26.2%) | 6/67 (9.0%) | 0.466 |
| Stroke history | 44/183 (24.0%) | 11/67 (16.4%) | 0.191 |
| Hypertension | 94/183 (51.4%) | 19/67 (28.4%) | 0.483 |
| Diabetes | 58/183 (31.7%) | 9/67 (13.4%) | 0.448 |
| Chronic kidney disease | 12/183 (6.6%) | 0/67 (0.0%) | 0.375 |
| Prior coronary angiography/PCI/CABG | 125/183 (68.3%) | 9/67 (13.4%) | 1.345 |
| Ezetimibe record near index | 38/183 (20.8%) | 8/67 (11.9%) | 0.24 |
| Documented concomitant statin therapy | 137/183 (74.9%) | 17/67 (25.4%) | 1.139 |
| Traceable baseline LDL-C available | 183/183 (100.0%) | 16/67 (23.9%) | 2.525 |

*Note. Eligibility-stage traceable LDL-C is shown only to describe cohort construction and selection; it is not the primary analysis baseline LDL-C definition used in Table 1.*

**Supplementary Table S2. Timing and number of pre-index LDL-C measurements**

| Group | N | Pre-index LDL-C measurements per patient, median (IQR) | Patients with >=2 pre-index LDL-C measurements | Closest pre-index LDL-C timing, days before index, median (IQR) | Earliest traceable pre-index LDL-C timing, days before index, median (IQR) | Any pre-index LDL-C within 30 days | Any pre-index LDL-C within 90 days | Any pre-index LDL-C within 365 days |
| --- | --- | --- | --- | --- | --- | --- | --- | --- |
| Overall | 183 | 2 (1 to 5) | 121/183 (66.1%) | 5 (2 to 29) | 175 (7 to 680) | 137/183 (74.9%) | 159/183 (86.9%) | 172/183 (94.0%) |
| Documented concomitant statin therapy | 137 | 2 (1 to 4) | 86/137 (62.8%) | 3 (2 to 10) | 112 (5 to 633) | 116/137 (84.7%) | 127/137 (92.7%) | 132/137 (96.4%) |
| No documented concomitant statin therapy | 46 | 3 (2 to 5) | 35/46 (76.1%) | 34 (9 to 122) | 279 (95 to 736) | 21/46 (45.7%) | 32/46 (69.6%) | 40/46 (87.0%) |

**Supplementary Table S3. Sensitivity analyses using alternative baseline LDL-C definitions**

| Baseline definition | Group | Paired N | Baseline LDL-C, median (IQR), mmol/L | 30-120 day follow-up LDL-C, median (IQR), mmol/L | Absolute change, median (IQR), mmol/L | Percent change, median (IQR) |
| --- | --- | --- | --- | --- | --- | --- |
| Primary: patient-level median of all traceable pre-index LDL-C values | Overall | 117 | 2.85 (2.36 to 3.75) | 1.08 (0.7 to 1.57) | -1.63 (-2.48 to -1.09) | -63.3 (-78.4 to -42.4)% |
| Primary: patient-level median of all traceable pre-index LDL-C values | Documented concomitant statin therapy | 94 | 2.83 (2.34 to 3.73) | 0.95 (0.68 to 1.37) | -1.74 (-2.61 to -1.14) | -67 (-79.7 to -47.2)% |
| Primary: patient-level median of all traceable pre-index LDL-C values | No documented concomitant statin therapy | 23 | 2.98 (2.42 to 3.63) | 1.42 (1.1 to 2) | -1.19 (-2.06 to -1) | -44.8 (-62.9 to -33.7)% |
| Median of pre-index LDL-C values within 365 days | Overall | 113 | 2.85 (2.26 to 3.63) | 1.08 (0.7 to 1.57) | -1.63 (-2.47 to -1.09) | -61.3 (-76.7 to -43.4)% |
| Median of pre-index LDL-C values within 365 days | Documented concomitant statin therapy | 91 | 2.84 (2.24 to 3.65) | 0.96 (0.68 to 1.36) | -1.72 (-2.51 to -1.19) | -63.5 (-79.2 to -51.9)% |
| Median of pre-index LDL-C values within 365 days | No documented concomitant statin therapy | 22 | 2.96 (2.37 to 3.46) | 1.47 (1.09 to 2.01) | -1.18 (-1.81 to -0.67) | -43.6 (-62.1 to -31.2)% |
| Closest pre-index LDL-C value within 90 days | Overall | 107 | 2.61 (2.07 to 3.71) | 1.05 (0.69 to 1.56) | -1.38 (-2.51 to -0.92) | -63.6 (-75.6 to -38.1)% |
| Closest pre-index LDL-C value within 90 days | Documented concomitant statin therapy | 89 | 2.69 (2.07 to 3.77) | 0.94 (0.68 to 1.34) | -1.54 (-2.56 to -0.99) | -65 (-77.3 to -39.9)% |
| Closest pre-index LDL-C value within 90 days | No documented concomitant statin therapy | 18 | 2.48 (2.15 to 3.1) | 1.61 (1.12 to 2.01) | -1.07 (-1.45 to -0.51) | -37.5 (-63.2 to -30)% |
| Closest pre-index LDL-C value within 30 days | Overall | 97 | 2.69 (2.07 to 3.77) | 1.03 (0.68 to 1.51) | -1.49 (-2.52 to -0.94) | -63.9 (-75.4 to -39)% |
| Closest pre-index LDL-C value within 30 days | Documented concomitant statin therapy | 83 | 2.73 (2.06 to 3.81) | 0.96 (0.67 to 1.33) | -1.54 (-2.55 to -0.99) | -64.8 (-78.2 to -40.3)% |
| Closest pre-index LDL-C value within 30 days | No documented concomitant statin therapy | 14 | 2.48 (2.15 to 3.06) | 1.51 (1.09 to 1.96) | -1.07 (-1.45 to -0.51) | -40.5 (-66.6 to -31.2)% |

**Supplementary Table S4. Baseline characteristics by 30-120-day LDL-C follow-up availability**

| Characteristic | With 30-120 day LDL-C follow-up (n=117) | Without 30-120 day LDL-C follow-up (n=66) | SMD |
| --- | --- | --- | --- |
| Age, years | 60 (60 to 70) | 60 (60 to 70) | -0.076 |
| Male sex | 79/117 (67.5%) | 36/66 (54.5%) | 0.268 |
| Documented concomitant statin therapy | 94/117 (80.3%) | 43/66 (65.2%) | 0.346 |
| Baseline LDL-C, mmol/L | 2.85 (2.36 to 3.75) | 2.77 (2.3 to 3.37) | 0.19 |
| Pre-index LDL-C measurements per patient | 3 (1 to 5) | 2 (1 to 3) | 0.347 |
| Closest pre-index LDL-C timing, days before index | 3.59 (1.59 to 13.56) | 13.77 (2.32 to 66.19) | -0.453 |
| Coronary artery disease-related evidence | 109/117 (93.2%) | 54/66 (81.8%) | 0.348 |
| Myocardial infarction history | 32/117 (27.4%) | 16/66 (24.2%) | 0.071 |
| Stroke history | 27/117 (23.1%) | 17/66 (25.8%) | -0.062 |
| Hypertension | 63/117 (53.8%) | 31/66 (47.0%) | 0.138 |
| Diabetes | 42/117 (35.9%) | 16/66 (24.2%) | 0.256 |
| Prior PCI | 70/117 (59.8%) | 29/66 (43.9%) | 0.322 |
| Prior coronary angiography/PCI/CABG | 83/117 (70.9%) | 42/66 (63.6%) | 0.156 |
| Ezetimibe record near index | 28/117 (23.9%) | 10/66 (15.2%) | 0.223 |
| Post-index observation span, days | 474 (293 to 661) | 395 (239 to 604) | 0.26 |

**Supplementary Table S5. Predictors of 30-120-day LDL-C follow-up availability**

| Predictor | Odds ratio | 95% CI | P value | Note |
| --- | --- | --- | --- | --- |
| Documented concomitant statin therapy | 1.83 | 0.82 to 4.12 | 0.141 | Multivariable logistic regression; diagnostic model for laboratory follow-up availability. |
| Age, per 10 years | 0.85 | 0.59 to 1.23 | 0.392 | Multivariable logistic regression; diagnostic model for laboratory follow-up availability. |
| Male sex | 1.59 | 0.75 to 3.36 | 0.227 | Multivariable logistic regression; diagnostic model for laboratory follow-up availability. |
| Baseline LDL-C, per 1 mmol/L | 1.6 | 1.08 to 2.37 | 0.019 | Multivariable logistic regression; diagnostic model for laboratory follow-up availability. |
| Diabetes | 2.11 | 0.93 to 4.78 | 0.075 | Multivariable logistic regression; diagnostic model for laboratory follow-up availability. |
| Coronary artery disease-related evidence | 3.42 | 0.92 to 12.78 | 0.067 | Multivariable logistic regression; diagnostic model for laboratory follow-up availability. |
| Prior coronary angiography/PCI/CABG | 0.71 | 0.29 to 1.76 | 0.460 | Multivariable logistic regression; diagnostic model for laboratory follow-up availability. |
| Ezetimibe record near index | 1.21 | 0.49 to 2.93 | 0.681 | Multivariable logistic regression; diagnostic model for laboratory follow-up availability. |
| Pre-index LDL-C measurements, per one measurement | 1.13 | 1 to 1.28 | 0.048 | Multivariable logistic regression; diagnostic model for laboratory follow-up availability. |
| Closest pre-index LDL-C timing, per 30 days farther from index | 0.9 | 0.83 to 0.97 | 0.010 | Multivariable logistic regression; diagnostic model for laboratory follow-up availability. |

**Supplementary Table S6. Inverse-probability-of-observation weighted LDL-C sensitivity analysis**

| Outcome | Available paired N, documented statin/no documented statin | Observation-weighted documented statin estimate | Observation-weighted no documented statin estimate | Weighted difference (documented statin minus no documented statin) | Bootstrap 95% CI | Unit | Note |
| --- | --- | --- | --- | --- | --- | --- | --- |
| LDL-C percent change, 30-120 days | 94/23 | -60.816 | -46.038 | -14.778 | -24.53 to -3.93 | percentage points | Inverse probability of 30-120 day LDL-C observation weighting; not a substitute for randomized follow-up. |
| LDL-C absolute change, 30-120 days | 94/23 | -1.874 | -1.391 | -0.483 | -0.91 to 0 | mmol/L | Inverse probability of 30-120 day LDL-C observation weighting; not a substitute for randomized follow-up. |

*Note. Observation weighting addresses measured predictors of follow-up testing and does not replace protocolized follow-up.*

**Supplementary Table S7. Repeated-measures GEE summary for LDL-C percentage change**

| Model | Patients | Window-level observations | Working correlation | Covariates | Main use |
| --- | --- | --- | --- | --- | --- |
| GEE Gaussian model for repeated LDL-C percent change | 156 | 315 | Exchangeable | treatment group, follow-up window, group-by-window interaction, baseline LDL-C, age, sex | Sensitivity analysis for longitudinal LDL-C pattern; not a protocolized durability endpoint. |

*Note. The model used available patient-window LDL-C percentage changes.*

**Supplementary Table S8. Repeated-measures GEE coefficients for LDL-C percentage change**

| Model term | Estimate | Robust SE | 95% CI | P value | Interpretation |
| --- | --- | --- | --- | --- | --- |
| Intercept | -7.271 | 13.121 | -32.99 to 18.45 | 0.580 | Expected percent change in the reference group/window at covariate value zero after model scaling conventions. |
| C(window)[T.121-365 days] | 8.435 | 4.429 | -0.25 to 17.12 | 0.057 | Change in percent response at 121-365 days versus 30-120 days in the no documented statin reference group. |
| C(window)[T.>365 days] | 6.531 | 5.523 | -4.29 to 17.36 | 0.237 | Change in percent response beyond 365 days versus 30-120 days in the no documented statin reference group. |
| combo | -14.271 | 5.012 | -24.09 to -4.45 | 0.004 | Adjusted group difference at the 30-120-day reference window; negative values favor documented statin therapy. |
| combo:C(window)[T.121-365 days] | -0.817 | 5.426 | -11.45 to 9.82 | 0.880 | Additional documented statin vs no documented statin difference at 121-365 days compared with the 30-120-day difference. |
| combo:C(window)[T.>365 days] | -0.542 | 6.856 | -13.98 to 12.9 | 0.937 | Additional documented statin vs no documented statin difference beyond 365 days compared with the 30-120-day difference. |
| LDL_C_baseline | -6.868 | 1.399 | -9.61 to -4.13 | <0.001 | Association between baseline LDL-C and percent change. |
| age_at_index | -0.28 | 0.187 | -0.65 to 0.09 | 0.134 | Association between age and percent change. |
| male | -0.769 | 3.223 | -7.09 to 5.55 | 0.811 | Association between male sex and percent change. |

In the repeated-measures GEE sensitivity analysis, 156 patients contributed 315 patient-window observations across the 30-120-day, 121-365-day, and >365-day follow-up periods. At the 30-120-day reference window, the adjusted difference in LDL-C percentage change for documented statin versus no documented statin was -14.27 percentage points (95% CI, -24.09 to -4.45). Group-by-window interaction terms were not statistically significant, indicating no clear evidence that the between-group pattern differed across later follow-up periods. The complete three-window subset analysis was descriptive and restricted to patients with LDL-C measurements available in all three follow-up windows.

**Supplementary Table S9. Window-level LDL-C summaries used for the repeated-measures analysis**

| Window | Group | Patients | Follow-up LDL-C, median (IQR), mmol/L | LDL-C percent change, median (IQR) | Tests per patient in window, median (IQR) | Follow-up day, median (IQR) |
| --- | --- | --- | --- | --- | --- | --- |
| 30-120 days | Overall | 117 | 1.08 (0.7 to 1.57) | -63.3% (-78.4% to -42.4%) | 1 (1 to 2) | 67 (48 to 84) |
| 30-120 days | Documented concomitant statin therapy | 94 | 0.95 (0.68 to 1.37) | -67% (-79.7% to -47.2%) | 1 (1 to 2) | 66 (45 to 82) |
| 30-120 days | No documented concomitant statin therapy | 23 | 1.42 (1.1 to 2) | -44.8% (-62.9% to -33.7%) | 1 (1 to 2) | 78 (59 to 99) |
| 121-365 days | Overall | 124 | 1.33 (0.81 to 1.9) | -53.3% (-70.1% to -31%) | 2 (1 to 3) | 231 (193 to 273) |
| 121-365 days | Documented concomitant statin therapy | 91 | 1.19 (0.74 to 1.7) | -58.5% (-74.2% to -40.5%) | 2 (1 to 3) | 229 (184 to 269) |
| 121-365 days | No documented concomitant statin therapy | 33 | 1.73 (1.3 to 2.38) | -41.9% (-56.6% to -23.2%) | 2 (1 to 3) | 232 (210 to 281) |
| >365 days | Overall | 74 | 1.37 (0.91 to 2) | -51.8% (-64% to -36.5%) | 2 (1 to 4) | 505 (429 to 585) |
| >365 days | Documented concomitant statin therapy | 52 | 1.12 (0.82 to 1.86) | -56.8% (-69.3% to -39.9%) | 2 (1 to 4) | 493 (419 to 567) |
| >365 days | No documented concomitant statin therapy | 22 | 1.76 (1.41 to 2.33) | -43.9% (-54% to -33.2%) | 2 (1 to 4) | 531 (476 to 608) |

*Note. Values are based on available measurements in each follow-up period.*

**Supplementary Table S10. LDL-C response among patients with measurements in all three follow-up periods**

| Group | N with LDL-C in all three windows | Baseline LDL-C | 30-120 days LDL-C | 30-120 days percent change | 121-365 days LDL-C | 121-365 days percent change | >365 days LDL-C | >365 days percent change |
| --- | --- | --- | --- | --- | --- | --- | --- | --- |
| Overall | 51 | 2.94 (2.38 to 3.69) | 1.1 (0.81 to 1.78) | -61.8% (-72% to -42%) | 1.42 (0.85 to 1.95) | -56.2% (-66.8% to -35.5%) | 1.36 (0.94 to 2.01) | -51.4% (-63.9% to -39%) |
| Documented concomitant statin therapy | 37 | 2.94 (2.4 to 3.52) | 1.01 (0.8 to 1.62) | -63.3% (-76.4% to -50.6%) | 1.26 (0.82 to 1.83) | -60.2% (-71.7% to -34%) | 1.19 (0.9 to 1.88) | -56.7% (-67% to -40.1%) |
| No documented concomitant statin therapy | 14 | 2.94 (2.4 to 3.98) | 1.47 (1.09 to 2.19) | -46.7% (-65.8% to -27.6%) | 1.69 (1.34 to 2.02) | -46.4% (-60.5% to -37.2%) | 1.76 (1.3 to 2.18) | -42% (-46.2% to -34.4%) |

*Note. This descriptive subset is not intended to represent the full cohort.*

**Supplementary Table S11. Endpoint-specific propensity-score balance diagnostics**

| Endpoint/sample | N, documented statin/no documented statin | Max absolute SMD before weighting | Max absolute SMD after weighting | Covariates with weighted SMD >0.10 |
| --- | --- | --- | --- | --- |
| LDL-C percent change, 30-120 days | 94/23 | 0.345 | 0.033 |  |
| LDL-C absolute change, 30-120 days | 94/23 | 0.345 | 0.033 |  |
| LDL-C <1.4 mmol/L, 30-120 days | 94/23 | 0.345 | 0.033 |  |
| LDL-C reduction >=50%, 30-120 days | 94/23 | 0.345 | 0.033 |  |
| Non-HDL-C percent change, 30-120 days | 94/23 | 0.345 | 0.033 |  |
| Lp(a) percent change, 30-120 days | 94/23 | 0.345 | 0.033 |  |
| sdLDL-C percent change, 30-120 days | 94/22 | 0.328 | 0.033 |  |
| CRP/hsCRP >=2 mg/L, all available post-index follow-up | 104/27 | 0.447 | 0.032 |  |
| Dual target: LDL-C <1.4 mmol/L and CRP/hsCRP <2 mg/L, all available post-index follow-up | 104/27 | 0.447 | 0.032 |  |
| hsCRP-TyG Delta CTI ANCOVA subset, 72h component window; 30-1095 days | 35/16 | Not applicable | Not applicable | ANCOVA endpoint; not analyzed using overlap weighting |
| CRP-TyG Delta CTI ANCOVA subset, 72h component window; 30-1095 days | 61/20 | Not applicable | Not applicable | ANCOVA endpoint; not analyzed using overlap weighting |

*Note. CTI models were analyzed using ANCOVA and are listed for transparency.*

**Supplementary Table S12. Propensity-score model specification**

| Covariate | Column | Type | Coding/transformation | Missing handling |
| --- | --- | --- | --- | --- |
| Age | age_at_index | continuous | standardized continuous | none |
| Male sex | male | binary | binary 0/1 | none |
| Coronary artery disease-related evidence | history_coronary_related_evidence | binary | binary 0/1 | none |
| ACS/unstable angina | history_ACS_or_UA | binary | binary 0/1 | none |
| Myocardial infarction history | history_MI | binary | binary 0/1 | none |
| Stroke history | history_stroke | binary | binary 0/1 | none |
| Hypertension | history_hypertension | binary | binary 0/1 | none |
| Diabetes | history_diabetes | binary | binary 0/1 | none |
| Chronic kidney disease | history_CKD | binary | binary 0/1 | none |
| Heart failure | history_HF | binary | binary 0/1 | none |
| Prior coronary angiography/PCI/CABG | prior_coronary_proc | binary | binary 0/1 | none |
| Ezetimibe record near index | ezetimibe_m30_p90 | binary | binary 0/1 | none |
| Baseline LDL-C | LDL_C_baseline | continuous | standardized continuous | none |
| Baseline non-HDL-C | non_HDL_C_baseline | continuous | standardized continuous | none |
| Baseline Lp(a) | Lp_a_baseline | continuous | standardized continuous | none |
| Baseline sdLDL-C | sdLDL_C_baseline | continuous | standardized continuous | median imputation (0.82) |
| Baseline sdLDL-C missing indicator | sdLDL_C_baseline_missing | binary | binary 0/1 | included because source covariate had missing values |

*Note. The lipid-adjusted model was used as a sensitivity analysis.*

**Supplementary Table S13. Propensity-score and overlap-weight diagnostics**

| Sample | Group | N | PS min | PS Q1 | PS median | PS Q3 | PS max | Overlap weight min | Overlap weight Q1 | Overlap weight median | Overlap weight Q3 | Overlap weight max | Overlap weight sum | Effective sample size |
| --- | --- | --- | --- | --- | --- | --- | --- | --- | --- | --- | --- | --- | --- | --- |
| Full 183-patient cohort | Documented concomitant statin therapy | 137 | 0.296 | 0.718 | 0.807 | 0.893 | 0.976 | 0.024 | 0.107 | 0.193 | 0.282 | 0.704 | 29.2 | 96.69 |
| Full 183-patient cohort | No documented concomitant statin therapy | 46 | 0.023 | 0.518 | 0.648 | 0.752 | 0.931 | 0.023 | 0.518 | 0.648 | 0.752 | 0.931 | 29.21 | 42.45 |

*Note. Effective sample size was calculated from overlap weights.*

**Supplementary Table S14. Exploratory CTI descriptive results**

| Analysis | CTI index | Group | N | Baseline CTI, median (IQR) | Post-index CTI, median (IQR) | Delta CTI, median (IQR) | Post-index day, median (IQR) | Baseline CTI panels, median (IQR) | Post-index CTI panels, median (IQR) |
| --- | --- | --- | --- | --- | --- | --- | --- | --- | --- |
| Main analysis | hsCRP-TyG | No documented statin | 16 | 4.992 (4.686 to 5.153) | 5.092 (4.523 to 5.283) | 0.127 (-0.156 to 0.225) | 408.894 (279.725 to 624.632) | 1.000 (1.000 to 2.000) | 1.000 (1.000 to 1.250) |
| Main analysis | hsCRP-TyG | Documented statin | 35 | 4.995 (4.474 to 5.529) | 4.526 (4.220 to 4.992) | -0.393 (-0.856 to 0.099) | 199.451 (72.021 to 326.733) | 1.000 (1.000 to 2.000) | 1.000 (1.000 to 1.000) |
| Main analysis | hsCRP-TyG | Overall | 51 | 4.995 (4.645 to 5.462) | 4.664 (4.262 to 5.163) | -0.123 (-0.638 to 0.161) | 270.023 (104.774 to 394.201) | 1.000 (1.000 to 2.000) | 1.000 (1.000 to 1.000) |
| Acute inflammation-excluded sensitivity | hsCRP-TyG | No documented statin | 15 | 4.988 (4.678 to 5.137) | 5.075 (4.456 to 5.254) | 0.121 (-0.195 to 0.240) | 473.392 (301.205 to 635.889) | 1.000 (1.000 to 2.000) | 1.000 (1.000 to 1.500) |
| Acute inflammation-excluded sensitivity | hsCRP-TyG | Documented statin | 35 | 4.995 (4.474 to 5.529) | 4.526 (4.220 to 4.992) | -0.393 (-0.856 to 0.099) | 199.451 (72.021 to 326.733) | 1.000 (1.000 to 2.000) | 1.000 (1.000 to 1.000) |
| Acute inflammation-excluded sensitivity | hsCRP-TyG | Overall | 50 | 4.992 (4.636 to 5.404) | 4.632 (4.255 to 5.138) | -0.142 (-0.641 to 0.135) | 276.491 (103.637 to 404.591) | 1.000 (1.000 to 2.000) | 1.000 (1.000 to 1.000) |
| Main analysis | CRP-TyG | No documented statin | 20 | 4.752 (4.567 to 5.419) | 4.810 (4.418 to 5.473) | 0.040 (-0.422 to 0.347) | 303.423 (220.976 to 400.019) | 2.000 (1.750 to 4.000) | 1.500 (1.000 to 3.000) |
| Main analysis | CRP-TyG | Documented statin | 61 | 4.914 (4.426 to 5.521) | 4.674 (4.268 to 5.120) | -0.298 (-0.656 to 0.081) | 278.543 (103.918 to 369.478) | 2.000 (1.000 to 3.000) | 1.000 (1.000 to 2.000) |
| Main analysis | CRP-TyG | Overall | 81 | 4.796 (4.469 to 5.514) | 4.737 (4.279 to 5.153) | -0.226 (-0.565 to 0.244) | 278.543 (137.393 to 386.416) | 2.000 (1.000 to 3.000) | 1.000 (1.000 to 3.000) |
| Acute inflammation-excluded sensitivity | CRP-TyG | No documented statin | 20 | 4.752 (4.567 to 5.419) | 4.810 (4.418 to 5.473) | 0.040 (-0.372 to 0.347) | 303.423 (220.976 to 398.532) | 2.000 (1.750 to 3.250) | 1.500 (1.000 to 3.000) |
| Acute inflammation-excluded sensitivity | CRP-TyG | Documented statin | 60 | 4.916 (4.456 to 5.515) | 4.580 (4.267 to 4.871) | -0.311 (-0.659 to 0.050) | 263.466 (101.678 to 358.335) | 2.000 (1.000 to 3.000) | 1.000 (1.000 to 2.000) |
| Acute inflammation-excluded sensitivity | CRP-TyG | Overall | 80 | 4.851 (4.471 to 5.501) | 4.705 (4.279 to 5.123) | -0.270 (-0.562 to 0.131) | 275.379 (133.795 to 360.028) | 2.000 (1.000 to 3.000) | 1.000 (1.000 to 2.000) |

*Note. CTI panels required component measurements within a 72-hour window.*

**Supplementary Table S15. Exploratory CTI ANCOVA models**

| Analysis | CTI index | Outcome | Model | N, documented statin/no documented statin | Adjusted difference (95% CI) | P value | Covariates |
| --- | --- | --- | --- | --- | --- | --- | --- |
| Main analysis | hsCRP-TyG | Delta CTI | ANCOVA | 35/16 | -0.423 (-0.749 to -0.097) | 0.011 | documented statin group, baseline CTI, age at index, male sex, diabetes history, baseline LDL-C |
| Main analysis | hsCRP-TyG | Delta CTI | ANCOVA + post-index day | 35/16 | -0.430 (-0.862 to 0.002) | 0.051 | documented statin group, baseline CTI, age at index, male sex, diabetes history, baseline LDL-C, post-index day |
| Acute inflammation-excluded sensitivity | hsCRP-TyG | Delta CTI | ANCOVA | 35/15 | -0.339 (-0.640 to -0.039) | 0.027 | documented statin group, baseline CTI, age at index, male sex, diabetes history, baseline LDL-C |
| Acute inflammation-excluded sensitivity | hsCRP-TyG | Delta CTI | ANCOVA + post-index day | 35/15 | -0.301 (-0.672 to 0.070) | 0.112 | documented statin group, baseline CTI, age at index, male sex, diabetes history, baseline LDL-C, post-index day |
| Main analysis | CRP-TyG | Delta CTI | ANCOVA | 61/20 | -0.247 (-0.564 to 0.071) | 0.128 | documented statin group, baseline CTI, age at index, male sex, diabetes history, baseline LDL-C |
| Main analysis | CRP-TyG | Delta CTI | ANCOVA + post-index day | 61/20 | -0.183 (-0.497 to 0.131) | 0.254 | documented statin group, baseline CTI, age at index, male sex, diabetes history, baseline LDL-C, post-index day |
| Acute inflammation-excluded sensitivity | CRP-TyG | Delta CTI | ANCOVA | 60/20 | -0.307 (-0.600 to -0.015) | 0.039 | documented statin group, baseline CTI, age at index, male sex, diabetes history, baseline LDL-C |
| Acute inflammation-excluded sensitivity | CRP-TyG | Delta CTI | ANCOVA + post-index day | 60/20 | -0.275 (-0.558 to 0.008) | 0.057 | documented statin group, baseline CTI, age at index, male sex, diabetes history, baseline LDL-C, post-index day |

*Note. Delta CTI was defined as post-index median CTI minus baseline median CTI.*

**Supplementary Table S16. Post-index laboratory abnormalities during follow-up.**

| Endpoint | Overall | Documented statin | No documented statin | P value |
| --- | --- | --- | --- | --- |
| ALT/AST >=3 x ULN | 13/165 (7.9%) | 10/124 (8.1%) | 3/41 (7.3%) | 1.0 |
| CK >=3 x ULN | 4/79 (5.1%) | 4/62 (6.5%) | 0/17 (0.0%) | 0.572 |

Values are n/N (%). Testing was not protocolized, and abnormalities were not adjudicated as evolocumab-related adverse reactions. ULN, upper limit of normal; ALT, alanine aminotransferase; AST, aspartate aminotransferase; CK, creatine kinase.

# Supplementary Figures

**Supplementary Figure S1. Repeated-measures GEE model for LDL-C percentage change**

*Note. The figure is provided as a supplementary diagnostic visualization.*

**Supplementary Figure S2. Propensity-score and overlap-weight distributions**

*Note. The figure is provided as a supplementary diagnostic visualization.*
